# Untapped potential of post‐exposure prophylaxis in sub‐Saharan Africa: a comparative analysis of PEP implementation planning in Kenya, Mozambique, Nigeria, Uganda and Zambia

**DOI:** 10.1002/jia2.26471

**Published:** 2025-06-26

**Authors:** Danielle Resar, Ambele Judith Mwamelo, Adebanjo Olowu, Janeen Drakes, Helder Macul, Eduarda de Gusmao, Julie Franks, Nere Otubu, Oluwakemi Osowale, Opeyemi Abudiore, Trevor Mwamba, Madaliso Silondwa, Prudence Haimbe, Hilda Shakwelele, Elo Otobo, Richard Borain, Marian Honu, Chidera Chizaram Igbomezie, Christopher Obermeyer, Tasha Vernon, Karin Hatzold, Heather Ingold, Michelle Rodolph, Sarah Yardly Jenkins

**Affiliations:** ^1^ Clinton Health Access Initiative Boston Massachusetts USA; ^2^ ICAP Global Health New York New York USA; ^3^ Clinton Health Access Initiative Abuja Nigeria; ^4^ Clinton Health Access Initiative Lusaka Zambia; ^5^ Children's Investment Fund Foundation London UK; ^6^ The Global Fund Geneva Switzerland; ^7^ Population Services International Cape Town USA; ^8^ World Health Organization Geneva Switzerland

**Keywords:** adolescent girls and young women, combination HIV prevention, differentiated service delivery, key populations, post‐exposure prophylaxis, sub‐Saharan Africa

## Abstract

**Introduction:**

In 2023, over 210,000 new HIV acquisitions occurred in Kenya, Mozambique, Nigeria, Uganda and Zambia. While uptake of oral pre‐exposure prophylaxis (oral PrEP) and coverage of voluntary medical male circumcision increased significantly over the past decade, post‐exposure prophylaxis (PEP) has received less attention and remains an underused HIV prevention intervention. In 2024, the World Health Organization (WHO) released new guidance emphasizing the need for timely access to PEP, including through community‐based channels and task‐sharing to mitigate barriers such as stigma and ensure timely access. We conducted a comparative analysis of PEP implementation planning to understand how PEP is currently integrated into HIV prevention programmes, and to identify barriers and opportunities for optimizing the impact of PEP in the method mix.

**Methods:**

We analysed Global Fund country proposals from Grant Cycle 6 (GC6) (2021−2023) and Grant Cycle 7 (GC7) (2024−2026) for five countries in Africa with high HIV burden and established PrEP programmes: Kenya, Mozambique, Nigeria, Uganda and Zambia. To understand how PEP implementation planning evolved across these two cycles, we used quantitative and qualitative analysis to identify trends. We extracted all PEP activities, coding them by focal population and activity type.

**Results:**

We found over a five‐fold increase in the number of PEP activities in GC7 compared to GC6, where there were only 10 PEP activities, and an expanded population focus, including people in prisons and pregnant and breastfeeding people. Proposals increasingly emphasized PEP not only as an intervention for occupational and sexual violence exposures but as a vital component of comprehensive HIV prevention strategies. Proposals described strategies for increasing access to PEP through differentiated service delivery models, including community‐led and pharmacy‐delivered approaches. However, PEP activities were not well defined, with PEP often included in product lists without articulating product‐specific activities to address barriers or increase access.

**Conclusions:**

All five countries demonstrated an increased focus on PEP from GC6 to GC7. While this reflects an ambition to expand access to PEP, product‐specific activities were not clearly articulated. Practical guidance and tools, as well as focused cross‐country learning to support the operationalization of WHO's recommendations, will be critical to increasing access and achieving impact.

## 1 INTRODUCTION

Despite progress in the HIV response in low‐ and middle‐income countries, access to HIV prevention remains inadequate. 1.3 million new HIV acquisitions globally in 2023 demonstrate that not enough people have access to acceptable, effective prevention [1]. Although programmes are increasingly scaling pre‐exposure prophylaxis (PrEP), the focus on post‐exposure prophylaxis (PEP) has been limited.

PEP was recommended by the World Health Organization (WHO) for occupational exposures in 2006 and then non‐occupational exposures in 2013 [2−5]. WHO released its first PEP‐specific guidelines in 2014 and most recently updated them in 2024 [6−8]. WHO's updated guidelines emphasize the need to support timely access to PEP, with recommendations for task‐shifting or sharing and community‐based delivery.

Despite WHO recommendations, there is limited research on PEP planning and implementation. There is also limited quantitative data available on use and impact, and no data tracked through the Global Fund or The President's Emergency Plan for AIDS Relief (PEPFAR) [9, 10]. Recent modelling estimating the impact of community PEP in Africa assumed current PEP use to be negligible [11]. While there is no data on the total addressable market, studies suggest significant unmet need. One study offering HIV prevention through online pharmacies in Kenya found PEP uptake was over seven times higher than PrEP uptake, despite the focus of demand creation on PrEP [12]. The study also found nearly all clients requested PrEP despite being recently exposed to HIV, suggesting low awareness of PEP [12].

In light of the new WHO guidelines and limited existing research, there is a need to take stock of how PEP has been integrated into prevention programmes. Countries are continuing to invest in PrEP, including differentiated and de‐medicalized service delivery. However, without a clear understanding of gaps and needs for PEP implementation, we risk missing an opportunity to maximize the impact of an intervention already widely available and highly affordable.

To assess trends in PEP implementation planning, we analysed Global Fund funding requests in Kenya, Mozambique, Nigeria, Uganda and Zambia. These countries accounted for 16% of new HIV acquisitions and >50% of people who received PrEP globally in 2023 [1, 9]. All five also have growing PrEP programmes—the number receiving PrEP increased by 40% from 2022 to 2023 [1, 9]. Based on increases in funding for PrEP and access to PrEP Matching Funds—an investment from the Children's Investment Fund Foundation (CIFF) to incentivize PrEP scale‐up through the Global Fund—we anticipate further PrEP expansion [13, 14]. Understanding PEP implementation planning trends within these high‐volume, growing prevention programmes may provide lessons for nascent markets. By focusing on five countries at the leading edge of prevention scale‐up, we aimed to identify lessons from PEP implementation planning that can inform HIV programmes more broadly in the region.

## 2 METHODS

We analysed publicly available funding requests for Global Fund Grant Cycle 6 (GC6) (2021−2023) and Grant Cycle 7 (GC7) (2024−2026) from Kenya, Mozambique, Nigeria, Uganda and Zambia [10]. Global Fund funding requests outline activities to be funded within the Global Fund allocation, serving as implementation plans for the 3‐year grant cycle. Governments develop funding requests through collaborative processes involving civil society, key and vulnerable populations, private sector, donors and technical partners, among others. While the Global Fund constitutes only a portion of overall HIV programming, these requests reflect national priorities and provide a basis for analysing implementation plans through a standard format [15]. The countries included in this analysis were selected based on their large existing PrEP programmes and eligibility for the CIFF‐Global Fund PrEP Matching Funds [14].

Quantitative analysis evaluated the frequency of PEP‐related activities within prevention modules of each funding request by extracting unique activities that referenced PEP, and coding activities by country and grant cycle. PEP activities were further categorized by type (service delivery, training and sensitization, commodity procurement, demand creation and awareness, policy and advocacy) and population. The data were analysed to obtain a descriptive summary of frequencies, trends, and differences by country and time period. We did not include mentions of PEP in other sections of the country proposals, such as above‐allocation requests to focus only on budgeted activities.

This study was a review of publicly available implementation plans and did not involve human subjects research.

## 3 RESULTS

**Figure 1:**
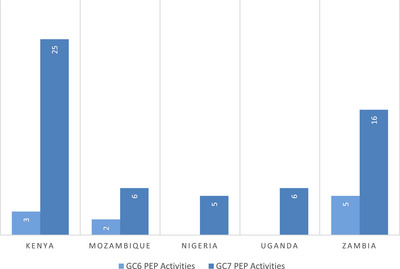
Number of PEP activities in funding requests by country and grant cycle. Abbreviations: GC6, Global Fund Grant Cycle 6; GC7, Global Fund Grant Cycle 7.

There was an increase in the focus on PEP between grant cycles, based on the activity count shown in Figure 1. In GC6, two countries (Nigeria and Uganda) did not include any PEP activities, while the other three countries (Kenya, Mozambique and Zambia) each included fewer than five. Across all five countries, the total number of PEP activities increased from 10 to 58 (over a five‐fold increase) from GC6 to GC7, with the greatest increase in Kenya, from three PEP activities in GC6 to 25 in GC7, a nearly eight‐fold increase.

Qualitative analysis of PEP activities found a broader population focus in GC7 compared to GC6, shown in Table 1. In GC6, PEP activities were focused within key population (KP) interventions, often within post‐violence services in drop‐in centres or one‐stop‐shops. In GC7, new population groups were included in PEP‐related activities. Kenya, Mozambique, Nigeria and Zambia included PEP activities for people in prisons and other closed settings. Uganda and Zambia included PEP for pregnant and breastfeeding people. Kenya, Mozambique and Zambia included PEP activities in prevention packages for a range of “other vulnerable populations,” including fisherfolk, serodiscordant couples, truckers and mobile populations. PEP programming in GC7 also had a stronger focus on interventions for adolescent girls and young women or adolescents and young people, with four countries (Kenya, Mozambique, Uganda and Zambia) including these populations in GC7 versus only two in GC6 (Kenya and Zambia). Uganda was the only country that did not include any PEP‐related activities in their package of services for men who have sex with men or transgender people in GC7, despite including a range of PrEP‐related activities for this population.

**Table 1:** Focal populations for PEP activities by country and grant cycle with example activities

Country	Grant cycle	Populations	Example activity
**Kenya**	GC6	• KPs: SWs, MSM, PWID, TGs, with an additional specific reference to young KPs • Healthcare workers	“Proposed activities under this intervention include Hepatitis B vaccination for healthcare workers; availability of PEP at all clinics and health centres; and availability of personal protective equipment at the facility level.”
	GC7	• KPs: SWs, MSM, PWID, TGs • People in prisons and other closed settings • AYP • Other vulnerable populations: fisherfolk, serodiscordant couples, truckers	“Capacity building of MSM‐led organizations on digital prevention approaches to improve demand creation for PrEP and PEP, event‐driven and other new prevention technologies.”
**Mozambique**	GC6	• KPs: SWs, MSM	“Offering post‐exposure prophylaxis according to national standards.”
	GC7	• KPs: SWs, MSM, TGP • People in prisons and other closed settings • AGYW • Other vulnerable populations: Truckers, internally displaced people, seasonal workers	“Screening, testing and treatment of asymptomatic STIs, including periodic serological testing for syphilis infection, delivery of cervical and anal cancer screening and linkages, emergency contraception, and PEP.”
**Nigeria**	GC6	*N/A—No PEP activities in GC6*	
	GC7	• KPs: SWs, MSM, TG, PWID • People in prisons and other closed settings	“Develop national prevention strategies, plans and programmes for access and uptake of HIV prevention tools including PrEP (various modalities) and PEP.”
**Uganda**	GC6	*N/A—No PEP activities in GC6*	
	GC7	• KPs: SWs, PWID • AGYW • Pregnant and breastfeeding people	“The country will also invest in provision of post‐violence counselling, referrals and linkages to PEP, clinical investigations, legal services, medical management, clinical care, and psychosocial support.”
**Zambia**	GC6	• KP: SWs, MSM, PWID, TGs • AYP	“Services at one stop centres include: HIV testing, emergency contraceptive, PEP and linkage to legal support for sexual violence victims, medical & surgical care with referral legal services for physically traumatized/assaulted victims, counselling to victims for relationships and psychosocial support.”
	GC7	• KPs: SWs, MSM, PWID, TGs • People in prisons and other closed settings • AYP • Pregnant and breastfeeding people • Other vulnerable populations: mobile populations, miners, truck drivers, orphans and vulnerable children, people with disabilities	“Build capacity of prisons HCWs and staff on delivery of prisoner‐friendly PrEP and PEP services.”

*Note*: Between grant cycles, there was a notable shift in activity type, moving towards broader integration of PEP into combination prevention packages, as shown in Figure 2. In GC6, PEP activities focused primarily on training, service delivery and commodity procurement. GC7 proposals demonstrated more structured, integrated approaches to implementation planning. For example, activities included key aspects for PEP delivery, such as demand creation, sensitization, and policy and advocacy. In GC7, PEP and PrEP were more frequently mentioned together, with both Kenya and Zambia including PEP alongside nearly every PrEP mention.

Abbreviations: AGYW, adolescent girls and young women; AYP, adolescents and young people; GC6, Global Fund Grant Cycle 6; GC7, Global Fund Grant Cycle 7; KPs, key populations; MSM, men who have sex with men; PEP, post‐exposure prophylaxis; PrEP, pre‐exposure prophylaxis; PWID, people who inject drugs; SWs, sex workers; TGs, transgender people.

**Figure 2:**
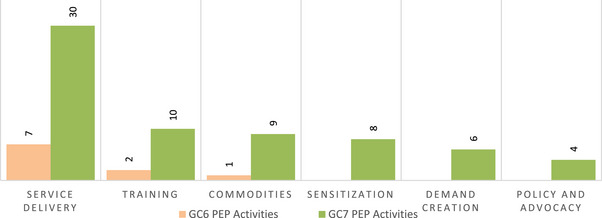
PEP activity type by grant cycle. *Notes*: Service delivery refers to the direct provision or offer of PEP. Training refers to capacity building and/or training provision to providers. Commodities refers to commodity procurement. Sensitization refers to providing information and education and building awareness to lay people, peers and/or healthcare providers who do not offer PEP but would link end users to PEP services. Demand creation refers to activities focused on generating demand for PEP. Policy and advocacy refers to activities to develop policies, strategies, plans or management structures to support PEP delivery. Abbreviations: GC6, Global Fund Grant Cycle 6; GC7, Global Fund Grant Cycle 7.

## 4 DISCUSSION

This analysis aimed to assess Global Fund PEP implementation planning across five countries with large, growing PrEP programmes. We observed a significant increase in the focus on PEP between grant cycles, across three dimensions investigated: number of PEP activities, populations and activity type. In GC7, PEP was treated as one intervention among a growing portfolio of prevention options and was often listed alongside various PrEP products. In contrast, GC6 PEP activities were primarily grouped within packages of care for post‐violence services or occupational exposures but not broader prevention packages. Increased focus on PEP alongside other PrEP products suggests that PEP may be benefiting from the growing commitment to expanding choice in prevention. This aligns with emerging themes in the literature—several recent studies investigated preferences or choice of PEP alongside PrEP options [11, 16−18].

Despite the increase in PEP‐related activities, few activities addressed product‐specific barriers or needs. While some activities referenced linkages between PEP and PrEP at a high level, funding requests did not clearly articulate the role of PEP in prevention packages. This aligns with a recent analysis of PEP policies across eight sub‐Saharan African countries, which found gaps and inconsistencies in how PEP is included in guideline and policy documents [19]. No country included the concept of “PEP‐in‐pocket” or referenced PEP as a prevention option among people with infrequent exposures in their funding requests, suggesting that these concepts have not been translated from research into implementation.

This analysis has key limitations. First, although Global Fund funding requests are developed collaboratively to reflect country priorities, they may not capture the full scope of programming in the country. Interventions funded by governments or other donors may be omitted from Global Fund funding requests to avoid duplication. Second, while funding requests are reviewed and approved by the Global Fund, activities may shift during implementation. This analysis did not evaluate how PEP activities were executed. This analysis also did not assess the budget allocated towards PEP—this is an area for future research. Next, although funding requests used a template that facilitated comparison across countries, some elements of the funding requests were flexible. For example, some countries grouped KP activities in a single module, while others included separate modules for each KP group. Grouping KP activities might result in fewer mentions of PEP but does not necessarily indicate lower prioritization. Finally, there is a critical gap in quantitative data on PEP use and no data available on PEP uptake from GC6 through the Global Fund. In addition, to date, countries have not set PEP targets as part of Global Fund implementation planning. Although PEP is referenced as part of prevention packages in Global Fund guidance, there are no PEP‐specific indicators included in the Global Fund's core list of indicators for HIV [20]. In 2023, both PEP and PrEP are listed as “HIV programme essentials” in Global Fund guidance for the first time, requiring countries to outline implementation progress [21]. However, the absence of PEP data presented in GC7 funding requests suggests there may be country‐level data gaps and with no Global Fund indicator for PEP use, it is unclear whether this data will be reported in the future. Similarly, there is no PEP data reported globally through UNAIDS or PEPFAR.

Without further analysis of other variables that impact PEP implementation, including budget, geographical coverage and targets, it is not possible to map the full scope of PEP programming across these countries. Moreover, with major shifts in foreign aid, including PEPFAR funding cuts, we may see greater variance in activities included in funding requests and interventions implemented, as governments prioritize immediate gaps. Even if a greater focus on PEP in funding requests translates to increased funding for PEP in this grant cycle, questions remain on sustainability amidst shrinking global health investments. In this context, PEP access may be more important than ever with treatment disruptions and reduced access to PrEP.

Despite limitations, this analysis highlights several clear implications and recommendations. First, PEP indicators should be included within the minimum set of HIV prevention indicators, guided by WHO's consolidated guidelines on person‐centred HIV strategic information, to support improved data visibility [22]. Programmes should also consider setting targets for PEP alongside planning for expanded access through differentiated service delivery, including community‐based delivery. Similarly, donors should include PEP indicators and targets in performance monitoring frameworks. While country‐specific implementation approaches will be critical, countries should also prioritize rapid evaluation of emerging data from pilots and other studies on the role of PEP within prevention portfolios, considering options like “PEP‐in‐pocket” as the focus on PEP grows beyond occupational exposures and sexual assault. Finally, with increasingly constrained funding, low‐resource strategies for PEP scale‐up are more critical than ever. This may include integrating PEP into self‐care packages and the use of HIV self‐tests to reduce provider burden and policy changes to permit PEP distribution among lower provider cadres.

## 5 CONCLUSIONS

All five countries analysed showed an increased focus on PEP in their Global Fund applications. While this reflects the growing ambition to expand access, product‐specific needs are not clearly articulated, suggesting the role of PEP within HIV prevention is not yet clearly understood or translated from research to implementation planning. Further evidence generation to define the added value of PEP and its optimal role within the prevention method mix can support countries in increasing access and impact more effectively. While choice‐focused, portfolio‐based approaches are essential for ensuring person‐centred services, it is equally important to identify and address barriers to timely PEP access, as well as to create demand and clearly communicate its benefit. Practical guidance and tools, as well as focused cross‐country learning to support the operationalization of WHO PEP recommendations, will be critical to addressing gaps and achieving impact to reduce new HIV acquisitions. Global organizations, such as the Global Fund and PEPFAR, should also consider strengthening data systems on PEP use to better understand need, coverage and impact.

## COMPETING INTERESTS

The authors declare no competing interests.

## AUTHORS’ CONTRIBUTIONS

DR, AO, AJM, JD and SYJ contributed to the study design, literature search and analysis. KH, TV, JF, HM, EG, MH, CO, CCI, NO, OO, EO, RB, OA, TM, PH, HS, MS, HI and MR contributed to manuscript drafting and review. All authors contributed to the study completion.

## FUNDING

This work has been funded by the Children's Investment Fund Foundation (CIFF). Publication fees will be paid by the supplement sponsor, the Gates Foundation.

## Data Availability

The data that support the findings of this study are openly available at: https://data.theglobalfund.org/
